# Substantia Nigra Radiomics Feature Extraction of Parkinson’s Disease Based on Magnitude Images of Susceptibility-Weighted Imaging

**DOI:** 10.3389/fnins.2021.646617

**Published:** 2021-05-31

**Authors:** Qingguo Ren, Yihua Wang, Shanshan Leng, Xiaomin Nan, Bin Zhang, Xinyan Shuai, Jianyuan Zhang, Xiaona Xia, Ye Li, Yaqiong Ge, Xiangshui Meng, Cuiping Zhao

**Affiliations:** ^1^Radiology, Qilu Hospital, Cheeloo College of Medicine, Shandong University, Qingdao, China; ^2^Neurosurgery, Qilu Hospital, Cheeloo College of Medicine, Shandong University, Qingdao, China; ^3^Radiology, Qingdao Municipal Hospital, Qingdao, China; ^4^Neurology, Qilu Hospital, Cheeloo College of Medicine, Shandong University, Qingdao, China; ^5^GE Healthcare, Shanghai, China

**Keywords:** Parkinson’s disease, magnetic resonance imaging, machine learning, substantia nigra, neuropsychological tests

## Abstract

**Background:**

It is reported that radiomic features extracted from quantitative susceptibility mapping (QSM) had promising clinical value for the diagnosis of Parkinson’s disease (PD). We aimed to explore the usefulness of radiomics features based on magnitude images to distinguish PD from non-PD controls.

**Methods:**

We retrospectively recruited PD patients and controls who underwent brain 3.0T MR including susceptibility-weighted imaging (SWI). A total of 396 radiomics features were extracted from the SN of 95 PD patients and 95 non-PD controls based on SWI. Intra-/inter-observer correlation coefficients (ICCs) were applied to measure the observer agreement for the radiomic feature extraction. Then the patients were randomly grouped into training and validation sets in a ratio of 7:3. In the training set, the maximum correlation minimum redundancy algorithm (mRMR) and the least absolute shrinkage and selection operator (LASSO) were conducted to filter and choose the optimized subset of features, and a radiomics signature was constructed. Moreover, radiomics signatures were constructed by different machine learning models. Area under the ROC curves (AUCs) were applied to evaluate the predictive performance of the models. Then correlation analysis was performed to evaluate the correlation between the optimized features and clinical factors.

**Results:**

The intro-observer CC ranged from 0.82 to 1.0, and the inter-observer CC ranged from 0.77 to 0.99. The LASSO logistic regression model showed good prediction efficacy in the training set [AUC = 0.82, 95% confidence interval (CI, 0.74–0.88)] and the validation set [AUC = 0.81, 95% CI (0.68–0.91)]. One radiomic feature showed a moderate negative correlation with Hoehn-Yahr stage (*r* = −0.49, *P* = 0.012).

**Conclusion:**

Radiomic predictive features based on SWI magnitude images could reflect the Hoehn-Yahr stage of PD to some extent.

## Introduction

Parkinson’s disease (PD) is the second most common neurodegenerative disease and affects 1% of the population above 60 years (Tysnes and Storstein, 2017). The main pathological change of PD is the degeneration and death of dopaminergic neurons in the substantia nigra (SN). Postmortem study has shown that loss of dopaminergic neurons occurs most severely in the lateral ventral tier, followed by the medial ventral tier of the SN pars compacta (Fearnley et al., 1991). Neuroimaging has a limited role in the diagnosis of PD especially in routine MR. In an *in vivo* study, susceptibility-weighted imaging (SWI) was found to be more sensitive for detection brain mineralization than conventional MRI sequences. After the pioneering work by Kwon et al., using SWI with ultrahigh field MRI (Kwon et al., 2012), the observations were reproduced by 3 T scanners, and all these studies focused on the subregion in the dorsal aspect of the SN with the loss of the “swallow tail” sign in PD patients (Kim et al., 2019). The inconsistency of the swallow tail sign occurrence in healthy subjects (Schmidt et al., 2017), and the disappearance of the swallow tail sign can also be found in some cognitive disorders (Rizzo et al., 2019), these findings make it more difficult to diagnose Parkinson’s disease using only MRI signal changes.

During the past decade, radiomics have developed rapidly not only in oncology studies but also for many other diseases. Radiomics are the processes for high-throughput extraction of quantitative features that result in the conversion of images into mineable data and the subsequent analysis of these data for decision support. These are different to the traditional practices of treating medical images as pictures intended solely for visual interpretation, and potentially improve diagnostic, prognostic, and predictive accuracy (Gillies et al., 2016). Recent study showed that some radiomics features extracted from quantitative susceptibility mapping (QSM) had promising clinical value for the diagnosis of PD (Xiao et al., 2019). But the preprocess of QSM is complicated and the easily recognizable pointers for the slice selection is not very clear. In SWI, the magnitude image can be acquired directly and has been an easy applicable diagnostic tool for nigral degeneration in PD (Schwarz et al., 2014). Whether the radiomics features based on magnitude image could help to distinguish PD from non-PD controls and the relationship between the clinical features and radiomics features has not reported before.

## Materials and Methods

### Data Collection

We recruited PD patients from the Movement Disorder Center at the neurological department of Qilu Hospital of Shandong University (Qingdao) from January 2016 to November 2019 who had undergone 3 T brain MR imaging including a SWI sequence. The study was performed in accordance with the code of ethics of the World Medical Association (Declaration of Helsinki) for experiments involving humans and the protocol was approved by the Research Ethic Committee of our hospital. Clinical diagnoses of PD were made according to established criteria (Postuma et al., 2015) by two specialists (CZ and JZ) who have specialized in movement disorders for more than 10 years. As a control group, we selected previously retrieved patients from the picture archiving and communication systems (PACS) with the following inclusion criteria: patients with diagnosis of headache or vertigo syndrome who had an MRI with SWI but without PD. The exclusion criteria of PD and control group were as follows: (1) acute cerebral infarction and hemorrhage; (2) a history of stroke, brain surgery and head trauma, or with obvious hypo-intensity lesions in the basal ganglia and brainstem in SWI; (3) brain tumor, cerebral inflammatory diseases (4) not scanned at the same 3.0 T MR; (5) an obvious artifact in SWI.

Firstly, we retrieved 1,015 consecutive cases of neurological patients who had undergone SWI scanning of which a total of 182 patients had a diagnosis of PD, and 38 of these 182 patients were excluded according to the exclusion criteria. Among the 144 PD patients, 54 patients had a complete evaluation including a series of neuropsychological tests and an MDS-UPDRS (movement disorder society-unified Parkinson’s disease rating scale) evaluation at the same period as the SWI scanning. Then, we filtered out 95 inpatients as the control group. In order to keep the number in the PD group equal to the control group, we randomly selected 41 patients among the other 90 PD patients who did not have evaluation scales. At last, 95 PD patients and 95 non-PD controls were incorporated into this study. Then, through the ratio of 7:3, 126 patients were randomly assigned to the training set (PD: *n* = 68, controls: *n* = 58) and 64 patients were assigned to the validation set (PD: *n* = 27, controls: *n* = 37).

### Scanning Parameters

All the SWI sequences were scanned using the 3 Tesla MRI system (Ingenia scanner, Philips Medical Systems, Netherlands). The axial scans were set parallel to the intercommissural line. Slice thickness = 2 mm; TR = 20 ms; TE = 27 ms; flip angle 15°; FOV = 220 mm; number of signal acquisition 1; and matrix size 284 × 230.

### Region of Interest Segmentation and Radiomic Feature Extraction

**FIGURE 1:**
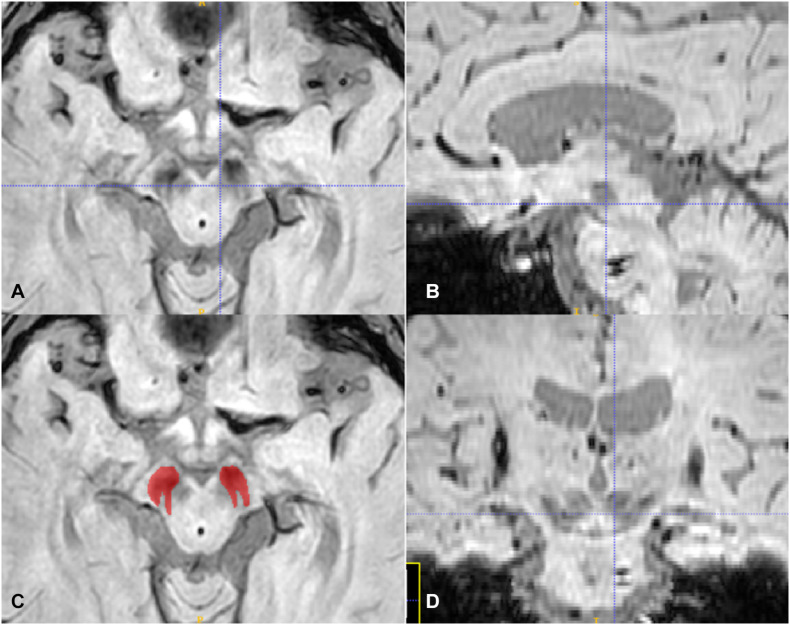
The axis **(A,C)**, sagittal **(B)**, and coronal **(D)** plane of the SN, the crossline located at the middle area of the red nucleus in **(A)** and at the bottom of the red nucleus in **(B,D)**. The red ROI in **(C)** is according to the SN hypo-intensity area in **(A)**.

We downloaded the original DICOM magnitude images of SWI sequences and imported them to the ITK-SNAP^1^ software for region of interest (ROI) drawing of the SN pars compacta. One experienced radiologist (QR) manual sketched the hypo-intensity area of the SN on the axis image at the level of the bottom of the red nucleus determined by the sagittal and coronal location, as was shown in Figure 1. After more than 1 month, the same radiologist performed a ROI drawing with the same method for intra-observer agreement assessment, and another radiologist (XS) performed ROI segmentation with the same method independently to assess inter-observer reliability. Intra-/inter-observer correlation coefficients (ICCs) were applied to measure the observer agreement for the radiomic feature extraction. ROIs with intra- and inter- observer CCs ≥ 0.75 were used for feature extraction.

We extracted radiomic features from the 2D slices of the SN ROI by AK software (Artificial Intelligence Kit V3.0.0.R, GE Healthcare); a total of 396 features were extracted including: (1) histogram features (*n* = 42), describing the intensity information and its overall distribution of the focus; (2) form factor features (*n* = 15), describing the shape, compactness, and other information of the focus by mathematical method; (3) gray level co-occurrence matrix (GLCM) features (*n* = 154), describing the complexity of the focus, the level change, and the texture thickness; (4) run length matrix (RLM) features (*n* = 180), and (5) gray level size zone matrix (GLSZM) features (*n* = 11), describing the complexity of the focus, the level change, the thickness of the texture, and other information.

### Feature Selection and Model Construction

The maximum correlation minimum redundancy algorithm (mRMR) was performed to eliminate the redundant and irrelevant features, and then, the least absolute shrinkage and selection operator (LASSO) was conducted to filter and choose the optimized subset of features in the training set. Moreover, radiomics signatures were constructed by different machine learning models including random forest (rf), linear support vector machine (svmLinear), radial support vector machine (svmRadial), and k nearest neighbors (knn) as well as logistic regression model. And the rad_score of each patient was calculated by selected features weighted by their coefficients by different machine models. According to each patient’s rad_score, the patients were classified into the PD and control class by an optimal cutoff value. The significant differences of the classification were evaluated by Wilcoxon test. We used ROC analysis to evaluate the performance of the model. Besides, to evaluate the stability of the best machine learning model, we performed 100-fold leave group-out cross-validation (LGOCV) analysis. Finally, we used a decision curve to evaluate the clinical usefulness of the model.

### Statistical Analysis

All statistical analyses were performed using R statistical software (v. 3.5.1)^2^. Firstly, the “mRMRe” package was used to screen the radiomic features for relevancy and non-redundancy. Then the LASSO logistic regression in the “Glmnet” package were applied to select the predictive features and construct the radiomics model. ROC curves were plotted using the “pROC” package. The “dca.R” package was applied to plot the decision curve.

The Spearman correlation analyzed these predictive features with clinical factors. The outcome results were interpreted according to the degree of association as strong (*| r|* >0.7), moderate (0.4<*| r|* <0.7), or mild (0.1<*| r|* <0.4) after taking significant correlation (*P* < 0.05) values into consideration.

## Results

### Demographic Characteristics

The clinical and demographic characteristics of the subjects are summarized in Table 1. There was no significant difference of age or sex between the two groups (*P* > 0.05).

**TABLE 1:** The demographic characteristic of the PD and control groups.

	PD	Control	*P*
Age (y, mean ± SD, range)	63.88 ± 10.08 (31–80)	62.53 ± 14.81 (19–85)	0.461
Gender (male/female)	44/51	47/48	0.663
Age of onset (y, mean ± SD, range)	58.42 ± 9.80 (30.5–75)	NA	
MDS-UPDRS	56.28 ± 26.45 (15–127)	NA	
MDS-UPDRS-I	11.94 ± 5.46 (1–22)	NA	
MDS-UPDRS-II	15.06 ± 8.13 (3–43)	NA	
MDS-UPDRS-III	27.46 ± 14.55 (8–62)	NA	
MDS-UPDRS-IV	2.04 ± 4.26 (0–19)	NA	
H&Y	1.85 ± 0.78 (1–4)	NA	
MMSE	27.33 ± 2.89 (17–30)	NA	
MoCA	19.73 ± 8.84 (10–29)	NA	
HAMA	9.70 ± 6.57 (0–28)	NA	
HAMD	15.28 ± 9.28 (1–42)	NA	
PDSS	115.56 ± 22.76 (66–149)	NA	
LEDD (mg)	491.51 ± 309.65 (75–1249)	NA	

*MDS-UPDRS, I-IV, Movement disorder society unified Parkinson’s disease rating scale, Part I–Part IV; H&Y, Hoehn-Yahr stage; MMSE, Mini-Mental State Examination; MoCA, Montreal Cognitive Assessment; HAHA, Hamilton Anxiety Scale; HAMD, Hamilton Depression Scale; PDSS, Parkinson disease sleep scale; LEDD, the equivalent levodopa daily dose was calculated according to previous study (Tomlinson et al., 2010).*

### Radiomics Signature Construction and Validation

**FIGURE 2:**
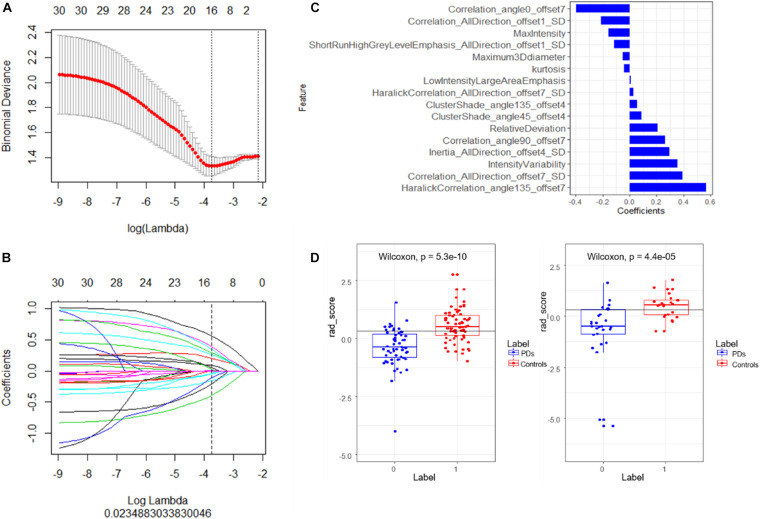
**(A)** Tuning parameter (λ) selection in the LASSO model using 10-fold cross-validation via minimum criteria. Binomial deviances from the LASSO regression cross-validation model are plotted as a function of log (λ). The coefficients vary by log (λ), the dotted vertical lines were drawn at the optimal values using the minimum criteria and the 1-SE criteria. **(B)** The LASSO coefficient profiles of 16 features with non-zero coefficients are shown in the plot. **(C)** The retained non-zero coefficients features are plotted on the *y*-axis and their coefficients in the LASSO Cox analysis are plotted on the *x*-axis. The rad-scores of each patient were calculated and classified into PD and control class according to the cutoff value 0.31. **(D)** Patients with a rad-score less than 0.31 represent the true classification of PD patients, bigger than 0.31 means PD patients were falsely classified into the control class. The Wilcoxon test shows there is a significant difference of the model in classifying the patients into these two classes.

At first, a total of 396 radiomic features were extracted from each subject. The intro-observer CC ranged from 0.82 to 1.0, and the inter-observer CC ranged from 0.77 to 0.99, which means the intro- and inter- observer were in good reproducibility, in the training set, the mRMR was performed and 30 features were retained. After that, a total of 16 PD-related features including five histogram features, one form factor feature, one RLM feature, and nine GLCM features were filtered to construct the radiomics signature using a multivariate logistic regression model (Figures 2A,B). The 16 features with non-zero coefficients are shown in Figure 2C and the weight of each feature that contributed to the established signature is displayed. According to the optimal cutoff value of 0.31, the patients were classified into PD class and control class (Figure 2D), there was a significant difference between the rad-scores in both the training (*p* < 0.001) and validation (*p* < 0.001) sets of the PD and control classes.

**FIGURE 3:**
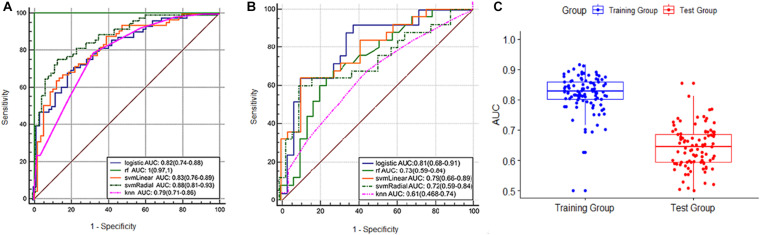
**(A)** ROC curve of the training set. **(B)** ROC curve of the validation set. **(C)** boxplot of the results of 100-fold LGOCV in the training and validation sets.

The radiomics signature showed good predictive performance with an AUC value of 0.82 (95% confidence interval (CI): 0.74–0.88) in the training set using the LASSO logistic regression model (Figure 3A), and the biggest value of 0.81 (95% CI: 0.68–0.91) in the validation set using the multivariate logistic regression model (Figure 3B). Based on the Youden index, accuracy, sensitivity, specificity, and other parameters were calculated, as shown in Table 2. In the training set, the accuracy, sensitivity, and specificity were 0.76, 0.69, and 0.81, and 0.69, 0.64, and 0.72 in the validation set, and the results were similar using different machine learning models in the validation set except for logistic VS knn, svmLinear VS knn, and svmRadial vs. knn (delong test, *p* < 0.05). rf was overfitted with an AUC of 1 (0.97–1) in the training group and 0.73 (0.59–0.84) in the test group, both multivariate logistic regressions, svmLinear and svmRadial showed preferable performance (Figure 3B). Besides, to verify the reliability of our results, we performed 100-fold LGOCV analysis in the multivariate logistic regression. The AUC values [mean ± standard deviation (SD)] of the 100 tests were 0.778 ± 0.191 and 0.606 ± 0.157 in LGOCV, respectively (Figure 3C).

**TABLE 2:** The statistical parameters of the training and validation set.

		Accuracy	Sensitivity	Specificity	Positive predictive value	Negative predictive value
Logistic	Train	0.76 (0.66–0.82)	0.69	0.81	0.81	0.70
	Validation	0.69 (0.54–0.80)	0.64	0.72	0.67	0.70
Rf	Train	1.00 (0.97–1.00)	1.00	1.00	1.00	1.00
	Validation	0.69 (0.54–0.80)	0.64	0.73	0.72	0.66
svmLinear	Train	0.75 (0.67–0.83)	0.86	0.68	0.64	0.88
	Validation	0.78 (0.64–0.88)	0.84	0.74	0.64	0.90
svmRadial	Train	0.81 (0.73–0.87)	0.86	0.76	0.76	0.86
	Validation	0.69 (0.54–0.80)	0.67	0.70	0.64	0.72
Knn	Train	0.74 (0.65–0.81)	0.74	0.74	0.79	0.68
	Validation	0.59 (0.45–0.72)	0.55	0.64	0.64	0.55

*rf, random forest; svm, support vector machine; knn, k nearest neighbors.*

**FIGURE 4:**
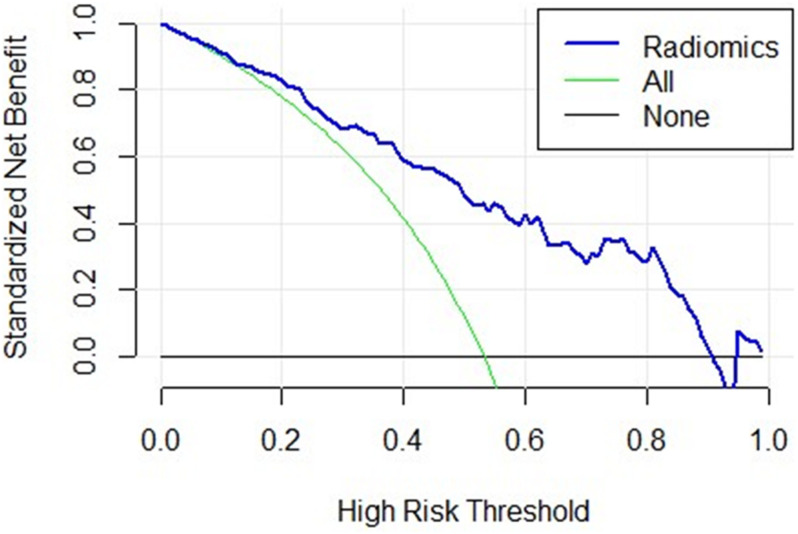
The decision curve of the radiomics signature. The *x*-axis represents the threshold probability, the *y*-axis represents the net benefit, the green curve represents the hypothesis that all patients were in the PD class, the black curve parallel to the *x*-axis represents the hypothesis that all patients were in the control class. The blue curve represents the threshold of 0.1–0.9, where the radiomics signature gains more benefit than treating all the patients or where no one was treated.

We used decision curves to evaluate the clinical usefulness of the model as shown in Figure 4. It shows that the radiomics signature was superior to the clinical model regarding “treat all” vs. “treat none” strategies when the threshold probability was within the 0.1–0.9 range.

### Correlation of the Predictive Features With Clinical Factor

**FIGURE 5:**
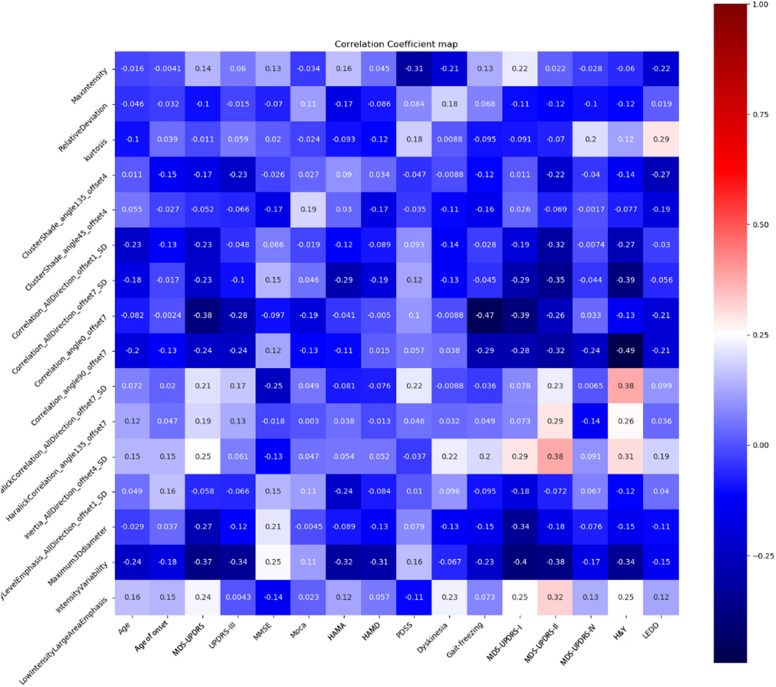
Correlation heatmap of the 16 significant features and clinical factors.

Spearman correlation analysis showed a moderate negative correlation between H&Y and correlation angle 90, offset 7 (*r* = –0.49, *P* = 0.012), gait freezing and correlation angle 0, offset 7 (*r* = –0.47, *P* = 0.0047), and UPDRS-I and intensity variability (*r* = –0.40, *P* = 0.006). There were another four radiomic features with correlation coefficient values of more than 0.3 between H&Y. The results are shown in Figure 5.

## Discussion

In this study, we investigated the changes of radiomic features of the SN based on SWI to differentiate PD from non-PD controls and the relationships between the radiomic feature and PD patients’ clinical characteristics. A radiomics signature constructed by 16 predictive features in our study showed good predictive efficacy in distinguishing PD from controls in the training set (AUC = 0.82, 95% CI: 0.75–0.89), and validated the validation set (AUC = 0.81, 95% CI: 0.70–0.93). Based on the Youden index, the sensitivity and specificity of the radiomics signature in the validation set were 64 and 72% for PD vs. controls, which was lower than a previous study (Cheng et al., 2019).

In a previous study, age was a significant determination of deep gray matter iron accumulation in normally aging subjects (Pirpamer et al., 2016). Not only affecting the normal subjects, the age of onset of PD may affect the patients’ phenotype, the rate of progression, and motor or non-motor complications (Palermo et al., 2020). What is more, the young-onset PD patients showed relatively less nigrostriatal degeneration than the late-onset ones (Kübler et al., 2019). Our results showed that more than a half of radiomics features with non-zero coefficients had no relation with age and the age of onset (| *r*| <0.1), and less than half of features only manifested mild correlations correspondingly. This result showed that age may be not a critical determination factor affecting the nigrostriatal radiomics feature.

Generally, the degeneration of the SN pars compacta is responsible for the appearance of the motor symptoms of PD patients (Mallet et al., 2019). It was also reported that iron deposition in the SN might be closely correlated with motor symptoms through neuroinflammation in PD patients (Liu et al., 2017). To our knowledge, there were few studies reporting the relationships between the SN and non-motor symptoms of PD. A recent report showed that a focused transcranial Doppler device targeting the SN had one or more improved cognitive scores in PD patients (Nicodemus et al., 2019). Although more than half of radiomics features with non-zero coefficients had no relation with MoCA, more than half had a mild relation with MMSE. We thought the SN could manifest the cognitive condition of PD patients to some extent.

Neuropsychiatric symptoms are now known to affect the majority of patients and contribute greatly to reduced quality of life in advanced PD patients. In a review by Butala et al. (2019), it was reported that the caudate nucleus, the dorsal raphe nucleus, the orbitofrontal cortex, and the limbic regions potentially contributed to more severe and refractory symptoms in depressed PD patients, while the brainstem nuclei, the anterior cingulate cortex, and the precuneus might relate to anxiety. Our results showed that most of the SN radiomic features had mild or even no correlation with psychiatric conditions, which was basically consistent with the above overview, with another possible reason that the traditional scales lack adequate specificity for psychiatric features in PD.

MDS-UPDRS included: I, non-motor experiences of daily living; II, questionnaire of daily living motor experiences; III, motor examination; IV, motor complication; and H&Y which related with disease progression. Our results showed more than half of radiomic features had a mild and moderate correlation with UPDRS, and separately with UPDRS-I, UPDRS-II, and H&Y. What is interesting, one radiomics feature (correlation angle 90, offset 7) had a moderate negative correlation with H&Y stage (*r* = −0.49, *P* = 0.012). This feature showed a linear dependency of gray level values to their respective voxels in the GLCM with angle = 90 and offset = 7, which is a value between 0 (uncorrelated) and 1 (perfectly correlated). Although more than half of the features had no correlation with gait freezing, one feature had a moderate correlation with gait freezing. Previous research based on MR multiple gradient echo sequences showed that gait-freezing PD patients had a greater motor score and nigral iron content than the non-freezing ones (Wieler et al., 2016). Moreover, deep brain stimulation of the SN pars reticulata has been reported to improve resistant gait freezing (Weiss et al., 2011). We thought the SN could indicate the severity of symptoms in Parkinson’s patients, including motor and non-motor symptoms to some extent.

In another aspect, our results showed that more than half of radiomic features had a mild correlation with LEDD. This result was the same as previous reports that the echogenic SN area by transcranial sonography correlated with dopamine reuptake (Weise et al., 2009), and magnetic susceptibility of the SN correlated with the LEDD (Langkammer et al., 2016). It may be speculated that the SN radiomic features obtained from SWI are able to detect tissue changes at a level that is primarily relevant for treatment response.

Several limitations in our primary and exploratory study should be noted: (a) we used a single slice of a 2D region of interest for radiomic feature extraction, (b) the sample size is relatively small because of the fact that this was a single-center study. Larger and more randomized samples are needed in the future.

In spite of these limitations, we constructed a fine model using SN radiomic features for distinguishing PD from non-PD controls and conducted analysis of the relationships between these features and clinical characteristics. What is encouraging is that our results indicated that radiomics based on SWI could provide the potential value of the prediction of PD progression.

## Data Availability Statement

The raw data supporting the conclusions of this article will be made available by the authors, without undue reservation.

## Ethics Statement

The studies involving human participants were reviewed and approved by the Research Ethic Committee of Qilu Hospital of Shandong University (Qingdao). Written informed consent for participation was not required for this study in accordance with the institutional requirements.

## Author Contributions

QR draft and submitted the manuscript. YW, XM, and CZ modified the manuscript. SL, XN, XS, XX, and YL collected the clinical data. YG made the statistical analysis. BZ finished the neuropsychological tests. JZ and CZ did the PD diagnose. All authors contributed to the article and approved the submitted version.

## Conflict of Interest

The authors declare that the research was conducted in the absence of any commercial or financial relationships that could be construed as a potential conflict of interest.

## Footnotes

1 http://www.itksnap.org/pmwiki/pmwiki.php

2 https://ww-w.Rproject.org

## Abbreviations

PD: Parkinson’s disease
SN: Substantia nigra
mRMR: Maximum correlation minimum redundancy algorithm
LASSO: Least absolute shrinkage and selection operator
PACS: Picture archiving and communication systems
MDS-UPDRS: Movement disorder society-unified Parkinson’s disease rating scale
GLCM: Gray level co-occurrence matrix
RLM: Run length matrix
GLSZM: Gray level size zone matrix
LGOCV: Leave group-out cross-validation
H&Y: Hoehn-Yahr stage
MMSE: Mini-Mental State Examination
MoCA: Montreal Cognitive Assessment
HAHA: Hamilton Anxiety Scale
HAMD: Hamilton Depression Scale
PDSS: Parkinson disease sleep scale
LEDD: Equivalent levodopa daily dose
CI: Confidence interval
SD: Standard deviation.
